# Economic Value of Vaccines to Address the COVID-19 Pandemic in Hong Kong: A Cost-Effectiveness Analysis

**DOI:** 10.3390/vaccines10040495

**Published:** 2022-03-23

**Authors:** Xuechen Xiong, Jing Li, Bo Huang, Tony Tam, Yingyi Hong, Ka-Chun Chong, Zhaohua Huo

**Affiliations:** 1 Institute of Future Cities, The Chinese University of Hong Kong, Hong Kong, China; xuechenxiong@cuhk.edu.hk (X.X.); victorli@cuhk.edu.hk (J.L.); 2 School of Public Health, Fudan University, Shanghai 200032, China; 3 Department of Geography and Resource Management, The Chinese University of Hong Kong, Hong Kong, China; 4 Shenzhen Research Institute, The Chinese University of Hong Kong, Shenzhen 518057, China; 5 Department of Sociology, The Chinese University of Hong Kong, Hong Kong, China; tony.tam@cuhk.edu.hk; 6 Department of Management, The Chinese University of Hong Kong, Hong Kong, China; yyhong@cuhk.edu.hk; 7 JC School of Public Health and Primary Care, The Chinese University of Hong Kong, Hong Kong, China; marc@cuhk.edu.hk (K.-C.C.); bobhuo@cuhk.edu.hk (Z.H.)

**Keywords:** vaccine, cost-effectiveness, economic analysis, Hong Kong

## Abstract

Objective The coronavirus disease 2019 (COVID-19) pandemic has imposed significant costs on economies. Safe and effective vaccines are a key tool to control the pandemic; however, vaccination programs can be costly. Are the benefits they bestow worth the costs they incur? The relative value of COVID-19 vaccines has not been widely assessed. In this study, a cost-effectiveness analysis was performed to provide evidence of the economic value of vaccines in Hong Kong. Method We developed a Markov model of COVID-19 infections using a susceptible–infected–recovered structure over a 1-year time horizon from a Hong Kong healthcare sector perspective to measure resource utilization, economic burden, and disease outcomes. The model consisted of two arms: do nothing and implement a vaccination program. We assessed effectiveness using units of quality-adjusted life years (QALYs) to measure the incremental cost-effectiveness at a HKD 1,000,000/QALY threshold. Results The vaccination program, which has reached approximately 72% of the population of Hong Kong with two vaccine doses, was found to have a cost of HKD 22,339,700 per QALY gained from February 2021 to February 2022. At a willingness-to-pay threshold, the vaccination program was not cost-effective in the context of the low prevalence of COVID-19 cases before the Omicron wave. However, the cost-effectiveness of a COVID-19 vaccine is sensitive to the infection rate. Hong Kong is now experiencing the fifth wave of the Omicron. It is estimated that the ICER of the vaccination program from February 2022 to February 2023 was HKD 310,094. The vaccination program in Hong Kong was cost-effective in the context of the Omicron. Conclusions Vaccination programs incur a large economic burden, and we therefore need to acknowledge their limitations in the short term. This will help relevant departments implement vaccination programs. From a longer-term perspective, the vaccination program will show great cost-effectiveness once infection rates are high in a regional outbreak. Compared with other age groups, it is suggested that the elderly population should be prioritized to improve the vaccine coverage rate.

## 1. Background

Severe acute respiratory syndrome coronavirus 2 (SARS-CoV-2) has been rapidly transmitted throughout the world. As of March 2022, there have been more than 438 million confirmed cases of coronavirus disease 2019 (COVID-19) globally, resulting in more than 5.9 million deaths since its onset in late 2019. The number of cases continues to increase rapidly. The worst-affected countries have been the United States (78.4 million cases, 0.95 million deaths), India (42.9 million cases, 0.51 million deaths), Brazil (28.8 million cases, 0.65 million deaths), France (22.2 million cases, 0.14 million deaths), and the UK (19.0 million cases, 0.16 million deaths) [1]. Hong Kong, as a densely populated international city with approximately 7.4 million people, has inevitably been severely affected by the pandemic. From January 2020 to March 2022, there have been approximately 748,919 confirmed cases of COVID-19 in Hong Kong, resulting in 4355 deaths [2]. The magnitude of morbidity and mortality associated with the pandemic, and its economic impact, have resulted in evolving global public health and economic crises [3]. In response, governments are urgently attempting to implement policies that slow and contain the COVID-19 pandemic [4,5].

### 1.1. Interventions against the COVID-19 Pandemic in Hong Kong

The Centre for Health Protection, Department of Health, Hong Kong, was established in 2004 [6]. With experience dealing with human swine influenza, avian influenza, and Middle East respiratory syndrome, Hong Kong gained considerable experience in coping with diseases potentially capable of progressing to pandemics [7,8]. With the outbreak of the COVID-19 pandemic in mainland China before Chinese New Year, the SAR government took measures to limit the movement of people. From 4 February 2020, travel between Hong Kong and mainland China and other regions was restricted [9]. On 29 March 2020, the “Prohibition on Group Gathering Regulation” was implemented [10]; on 15 July 2020, the “Wearing of Mask Regulation” was implemented [11]; on 15 November 2020, the “Compulsory Testing for Certain Persons Regulation” was implemented [12]; and on 22 December 2020, the “Designated Hotels for Quarantine” regulation was implemented [13]. Due to the implementation of these strict policies, the pandemic in Hong Kong has been effectively controlled [14,15]. However, these measures have inevitably caused substantial disruption to the entire society [16,17]. In the context of negotiations between social restrictions and epidemic control, safe and effective vaccines are becoming increasingly critical to end the COVID-19 pandemic and revive the economy [18]. Hong Kong once contained COVID-19 successfully, managing to keep infection rates among the lowest in the world. However, the Omicron variant spreads more easily than the original virus that causes COVID-19 and the Delta variant, even though the “Vaccination Pass” has been required since 24 February 2022 [19]. In view of the serious pandemic condition, the healthcare system of Hong Kong is under extreme strain. To prevent the collapse of the health system, the government began to arrange for close contacts and household contacts of close contacts who are deemed appropriate after assessment, to undergo home quarantine [20], so that the medical system can treat more patients with severe symptoms.

### 1.2. The Vaccination Program of Hong Kong

Social activities and economic function have been severely affected by the pandemic. Various industry sectors and the public are lobbying to relax social distancing measures and restore “normal” economic activity as soon as possible, on the basis of conditions such as a “vaccine bubble” [21]. Hong Kong’s vaccination program was officially launched on 26 February 2021, with the aim to safeguard public health and gradually allow the resumption of normal societal activities. Under the program, vaccines are provided free of charge to all Hong Kong residents. Currently, the government provides the public with two types of vaccines: an inactivated virus-based vaccine produced by Sinovac Biotech (Hong Kong) Limited and an mRNA-based vaccine produced by Fosun Pharma in collaboration with the German drug manufacturer BioNTech (BNT162b2 mRNA vaccine). Individuals are required to receive two doses of the same vaccine to achieve adequate protection. By March 2021, 90% of Hong Kong residents had received the first vaccine dose and 77% had received the second vaccine dose [22]. Furthermore, from 11 November 2021, the Hong Kong SAR government has encouraged eligible persons to receive a vaccine booster dose. Preliminary data suggested that Omicron may cause more mild disease; although the two-dose vaccination rate in Hong Kong is over 70%, the actual protection rate against infection with the variant virus Omicron is only about 16.5%. However, it is recognized that the vaccine provides insufficient protection against mild cases, but is still effective against severe cases and deaths [23]. In this regard, vaccine remains the best public health measure to protect people from COVID-19 and reduce the likelihood of new variants emerging.

### 1.3. A COVID-19 Vaccination Program May Be Expensive. Are the Benefits Worth the Costs?

Economies have already incurred significant costs due to the COVID-19 pandemic, causing increasing pressure on health budgets. It is essential that sufficient financial resources be guaranteed to implement vaccination programs, despite the substantial effort they represent [24]. Vaccines must be given to many individuals who may not otherwise contract the disease to achieve herd immunity through vaccination [25]. In addition, vaccinating a large number of individuals in a short timeframe requires large scale extra cost [26]. Vaccination’s socio-economic effects is receiving increasing attention [27,28,29]. Is vaccination cost effective in addressing COVID-19? The relative value of a vaccine for COVID-19 has not been widely assessed, especially in areas where the pandemic is comparatively controlled with strict measures.

Hong Kong has certain characteristics in implementing the vaccine program as it is transitioning from a low-infection area to a high-infection area. On one hand, Hong Kong adopted strict epidemic prevention measures early and succeeded in keeping the epidemic at a low level. Local epidemic levels in Hong Kong were low when the vaccine program was introduced. Whether widespread vaccination with low epidemic risk was cost-effective was an important question at the time. On the other hand, the Omicron variant virus spread around the world, and Hong Kong was not immune to the outbreak. Since 2022, Hong Kong has experienced a local outbreak of Omicron virus. Is it cost-effective to vaccinate widely when there is a high risk of epidemic transmission, and is it cost-effective to vaccinate when there is lower vaccine protection rate against patients with mild symptoms? This paper is focusing on answering these questions, which will help us to acknowledge the changing cost-effectiveness of Hong Kong’s vaccination program under different circumstances.

## 2. Methods

### 2.1. Study Design

We developed a Markov model to measure economic burden, and disease outcomes with or without vaccines [30]. A unidirectional susceptible–infected–recovered (SIR) structure was used to design the model. Once an individual is infected, they remain infected until they recover, worsen, or end the time horizon. The model was conducted on a 1-day cycle and results are reported over a 1-year time horizon.

We used the cost-effective analysis to evaluate the economic value of vaccines addressing the COVID-19 pandemic from the healthcare sector perspective. The incremental cost-effectiveness ratio (ICER) was calculated by dividing the incremental cost resulting from vaccination by measures of health outcomes to provide a ratio of the extra cost per extra unit of health effect. The ICER threshold depends on the willingness to pay. The World Health Organization suggests basing the threshold for the cost-effectiveness of interventions on the gross domestic product (GDP) [31]. Based on a GDP of USD 46,300 per capita for Hong Kong in 2020 [32], we set the willingness-to-pay threshold at HKD 1,000,000/QALY. In addition to ICER value used to reflect the cost-effectiveness of vaccination program, the Cohen’s D index was used as the effect size to compare the changes before and after the implementation of the vaccine program.

We considered scenarios with different infection rates. Scenario A reflected the actual spread of the pandemic when the vaccination program was launched, with an infection rate of 0.12%. Scenarios B–G assumed a spread of the pandemic with increasing infection rates from 0.25% to 2%. Scenarios H–K assumed increasing infection rates of 5%, 10%, 20%, and 30%, respectively, in the context of Omicron wave. We considered scenarios with different protection rates against patients with mild symptoms under Omicron wave. Scenarios L–O assumed a changing protection rates from 16.5% to 91.3%. We considered scenarios with different vaccination rate under Omicron wave. Scenarios P–Q assumed a changing vaccination rates from 72% to 90%. The model compared the vaccination program for the entire Hong Kong population with no vaccination, with comparisons made for the cost, the number of cases and deaths prevented, and the health utility gained.

### 2.2. Model Structure

**Figure 1:**
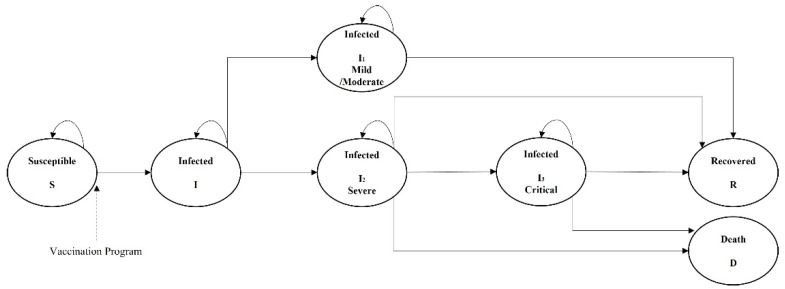
Markov model of COVID-19 disease progression.

The SIR transition model was used to simulate the transition of the Hong Kong population through seven mutually exclusive health states. At the end of each cycle, individuals were able to transition to other health states or remain in the current state, as indicated by the arrows in Figure 1. Vaccine efficacy was modeled as changing the transition pattern from susceptible to mild/moderate states, severe states, critical states, and death.

Once infected, the Department of Health arranges for admission to a public hospital for treatment as soon as possible. Individuals remained in the infected state until they worsened or the model ended. We assumed three separate infected states (I_1_–I_3_) based on current information about COVID-19 in Hong Kong. I_1_ indicated a mild or moderate infected state, without signs of severe pneumonia; I_2_ represented a severe disease state; and I_3_ represented a critical disease state [33]. According to Hong Kong’s intervention measures, once an individual is infected, they are hospitalized immediately, so all infected states require hospitalization, including mild and moderate states (I_1_). During hospitalization, as the course of the disease progresses, patients may progress to severe or critical states (I_2_). Patients who survived infection transitioned to the recovered (R) state, while those in severe or critical states (I_2_) had a high risk of death.

Each of the infection states had different periods of incubation prior to escalation or recovery. In I_1_, the median length of hospitalization for observation was 12 days for vaccinated patients and 15 days for unvaccinated patients [34]. In I_2_, patients who experienced acute respiratory distress syndrome needed additional medical intervention in an intensive care unit for 8 days [35]. Sixty-four percent of patients in I_2_ transferred to I_3_ and needed support from a ventilator or extracorporeal membrane oxygenation (ECMO). For patients with mild or moderate disease, the hospital stay lasted for 12–15 days. For patients with severe or critical disease, the hospital stay lasted for 20–23 days, including 8 days in the intensive care unit (ICU). After an individual completed their time in the infection states, they either recovered (R) or died (D).

### 2.3. Model Parameters

#### 2.3.1. Transition Probabilities

The COVID-19 outbreak in Hong Kong was first recorded in January 2020. Subsequently, the government gradually implemented strict measures to control it. The vaccination program was officially initiated in February 2021, while during the 1-year period from February 2020 to February 2021, the management of the pandemic was in a stage of non-vaccine intervention. Therefore, we set the spread of COVID-19 before February 2021 as the basic background of the pandemic under non-vaccine intervention and estimated basic transmission parameters. The basic infection and mortality rates were obtained from public data from the Hong Kong SAR government, although some data of patients with mild, severe, and critical disease were not available. We supplemented these data with information from the government’s official daily press conferences held whenever there were new cases. From February 2020 to February 2021, 10,927 cases were reported in Hong Kong. In the third wave of the pandemic in Hong Kong, among 4683 patients, 4484 had mild disease and 199 patients with severe disease were transferred to ICUs, of whom 64% were put on ventilators or required ECMO for survival. Seventy-five of the patients with severe disease died, with a mortality rate of 1.6%, and 124 recovered and were discharged from hospital [36]. The detailed transition probabilities are listed in Table 1.

#### 2.3.2. Vaccine Efficacy

Vaccine efficacy was modeled as the proportional reduction in the probability of SARS-CoV-2 infection. There are two vaccines available in Hong Kong: the CoronaVac COVID-19 Vaccine (Sinovac) and the Comirnaty COVID-19 mRNA Vaccine (BioNTech). Both of them have shown effectiveness against COVID-19 [37,38,39,40,41]. The BioNTech vaccine was shown to be 91.3% effective against COVID-19, as measured from 7 days to 6 months after the second dose. Clinical phase 3 trials in Brazil showed that the primary efficacy of CoronaVac (Sinovac) was 50.65% (95% confidence interval [CI]: 35.94–61.98). Numerous studies have reported that COVID-19 vaccine effectiveness decreases over time [42,43]. The Pfizer-BioNTech vaccine’s effectiveness against infection decreases from 86.9% to 43.3% after 6 months. In the modeling process, we set the effectiveness of both vaccines to remain stable up to 6 months and then decrease by half in the following 6 months. We also assumed that the efficacy of the vaccines was generally consistent across different age groups.

#### 2.3.3. Cost

Two components were used to evaluate cost. In addition to the direct cost of healthcare services, productivity loss was calculated separately. Infected patients were assigned the cost of a lost workday. In 2020, the median monthly salary of Hong Kong residents was HKD 18,200. This was divided by 30 days to give an average cost of HKD 600 per person per day [44]. For each day an individual was sick, society lost this amount [45].

The healthcare cost included polymerase chain reaction tests, hospitalization care, and ICU care. Patients in I_1_ were assigned daily costs for tests, hospitalization, and full sick leave. Patients in I_2_ were assigned daily costs for tests, medication, hospitalization, ICU care, and full sick leave. Patients in I_3_ were assigned daily costs for tests, medication, hospitalization, ICU care, respiratory machine treatment, and full sick leave. The test, hospitalization, and ICU care costs were obtained from various public price guidance documents. The costs of respiratory machine treatment and ECMO were not available, and we therefore assumed that these costs were the same as the cost of ICU care. Individuals in health states S and R were assumed to have no medical care costs or any additional social costs.

The costs of the vaccines were obtained from the Hong Kong budget for the influenza vaccine, with the target of having the majority of the population vaccinated for free in 2021 [46]. The government has earmarked more than HKD 8.4 billion for the procurement and administration of COVID-19 vaccines, which includes HKD 5.46 billion to buy at least two vaccines and HKD 2.9 billion for staff and administrative expenses to implement the program. Based on two doses of each type of vaccine, the cost of each dose per person was calculated to be HKD 369, with an additional HKD 196 for operational costs.

#### 2.3.4. Health Utilities

QALY is a universal measure of disease burden. QALY ranges from 0 to 1: QALY equals 0 for death and QALY equals 1 for full health. Utility losses reflect impaired health-related quality of life. Here, the utility losses were used for those people experiencing morbidity due to COVID-19. In addition, we assumed an average of 0.919 QALYs for the Hong Kong population [47].

**Table 1:** Model main parameters.

Parameter	Parameter	Based Value	References
Transition Parameters			
No vaccination Program	S to I	0.12%	[2]
	I to I_1_	95.75%	[36]
	I to I_2_	4.25%	
	I_2_ to I_3_	64.00%	
	I_1_ to R	100%	
	I_2_/I_3_ to R	62.35%	
	I_2_/I_3_ to D	37.65%	
Effect modification of Vaccine			
Probability of vaccinated		70.0%	[22]
	Age 3–11	1.8%	
	Age 12–19	66.9%	
	Age 20–59	86.2%	
	Age over 60	61.6%	
Sinovac	Probability of vaccinated by Sinovac	38.7%	[22]
	Primary Efficacy for mild case	83.70% [57.99–93.67%]	[41]
	Primary Efficacy for severe case	100% [56.4–100%]	
	Primary Efficacy for death	100% [56.4–100%]	Assumed
Biontech	Probability of vaccinated by Biontech	61.3%	[22]
	Primary Efficacy for mild case	91.3% [89.0–93.2%]	[38]
	Primary Efficacy for severe case	95.3% [71.0–100%]	
	Primary Efficacy for death	95.3% [71.0–100%]	Assumed
Cost and Utility Parameters			
Health care cost	Cost of Sinovac (per dose)	HKD 369	[46]
	Cost of Biontech (per dose)	HKD 369	
	Operation cost (per dose)	HKD 196	
	Cost of general ward/day	HKD 5100	[48]
	Cost of ICU/day	HKD 24400	
	Cost of Reverse transcription polymerase chain reaction (RT-PCR) Test	HKD 240	[49]
Productivity Loss	Loss of salary per person per day	HKD 600	[44]
Utility Loss	Health utility loss of S Susceptible	0.081	[47]
	Health utility loss of I_1_ Mild/moderate	0.50	[50]
	Health utility loss of I_2_	0.75	[51,52]
	Health utility loss of I_3_	0.95	[24]

### 2.4. Sensitivity Analysis

Given the uncertainty in estimating the effects of alternative inputs and assumptions, we conducted sensitivity analyses to test the model uncertainty by varying the expected parameter values [53]. Parameter value uncertainty was within ±25% of its expected value. To distinguish the difference in the overall effect of vaccination rate changes in different age groups, we divided the population into the following four age groups: 0–11, 12–19, 20–59, and above 60 years old. Increasing vaccination coverage is the goal of the vaccine program. An increase in vaccination coverage to 90 per cent was assumed for all age groups to assess differences in the cost-effectiveness of vaccination program. Therefore, in sensitivity analysis, the vaccination rate for each age group ranged from the current vaccination rates to 90%. In the sensitivity analysis, we use univariate one-way sensitivity analysis and Bayesian multivariate probabilistic sensitivity analysis (PSA). The PSA was performed using 100,000 Monte Carlo simulations, with no vaccination as the baseline approach.

## 3. Results

### 3.1. General Results

The pandemic situation in Hong Kong from January 2020 to February 2021 with an infection rate of 0.12% was set as the basic pandemic background of the vaccination program. Our model then simulated the cost and effectiveness of the vaccination program. For a 1-year period, the vaccination program cost approximately HKD 861 per person, compared with HKD 125 per person for no vaccination. In the Hong Kong population of 7.4 million people, the baseline approach, i.e., no vaccination program, was associated with a cost of HKD 0.8 billion, with 9630 infected cases and 269 deaths, while the vaccination program was associated with a cost of HKD 5.8 billion, with 3704 infected cases and 67 deaths. The vaccination program cost HKD 4.9 billion more than no vaccination, while preventing 5926 cases and 202 deaths and gaining 222 QALYs. The vaccination program, reaching approximately 72% of the population of Hong Kong with two doses of either the BioNTech or Sinovac vaccine per person, cost HKD 22,339,700 per QALY gained. Thus, at the willingness-to-pay threshold set for Hong Kong, the vaccination program in Hong Kong was not cost-effective in the context of the low prevalence of cases before the Omicron wave.

However, the Omicron variant spreads much more easily than the original virus that causes COVID-19, as well as the Delta variant. Under the wave of the Omicron epidemic, the infection rate in Hong Kong increased sharply and medical resources were scarce. We set Omicron scenarios with 10% average annual infection rate, adjusted protection rate against mild infection to 16.5%, and assumed that all patients with mild or moderate symptoms are quarantined at home to simulate the cost-effectiveness ratio under the Omicron wave. For a 1-year period, the vaccination program cost approximately HKD 1312 per person, compared with HKD 761 per person for no vaccination. In the Hong Kong population of 7.4 million people, the baseline approach, i.e., no vaccination program, was associated with a cost of HKD 5.1 billion in the first year alone, with 948,703 infected cases and 2,4043 deaths, while the vaccination program was associated with a cost of HKD 8.8 billion, with 827,682 infected cases and 9092 deaths. The vaccination program cost HKD 3.7 billion more than no vaccination, while preventing 121,021 cases and 14,951 deaths and gaining 11,967 QALYs. The vaccination program cost HKD 310,094 per QALY gained. Thus, at the willingness-to-pay threshold set for Hong Kong, the vaccination program in Hong Kong was cost-effective in the context of the Omicron wave. The detailed results of simulated ICER are listed in Table 2.

### 3.2. Sensitivity Analysis

**Figure 2:**
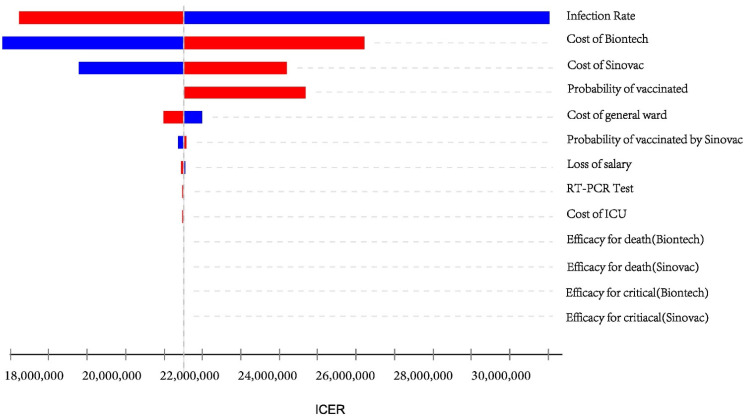
Tornado diagram of a one-way sensitivity analysis of parameter variabilities.

In sensitivity analysis, the incremental cost per QALY gained was most sensitive to changes in the infection rate, the vaccine price, the vaccination rate, and the cost of hospitalization (Figure 2).

The infection rate was one of the most sensitive variables affecting the cost-effectiveness of the vaccination program. When the basic infection rate increased, the ICER decreased, gradually approaching Hong Kong’s willingness-to-pay threshold. The following scenario analysis will detail this. The cost of the vaccine is another sensitive variable, with higher prices resulting in a higher ICER. Furthermore, Hong Kong supplies two types of vaccines with different coverage rates and efficiencies. A comparison of these two vaccines revealed that the BioNTech vaccine was more sensitive than the Sinovac vaccine at the same price and vaccination rate variation.

**Figure 3:**
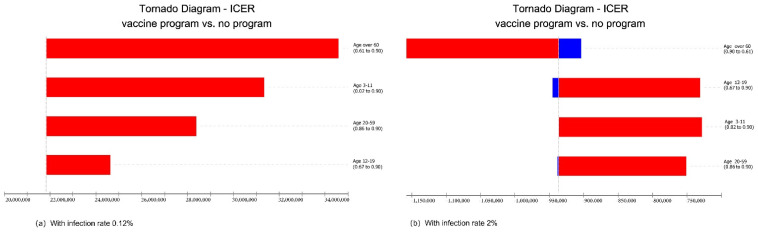
Tornado diagram of a one-way sensitivity analysis of vaccination rates by age group.

Changes in vaccination rates for different age groups from the current rate to 90% had different effects on the vaccination program (Figure 3). Under the current low infection rate (0.12%) in Hong Kong, increasing the vaccination rate in any age group brought a greater cost and higher ICER values. When the infection rate increased to 2%, an increase in vaccination rates was more cost-effective for older people than for other age groups.

### 3.3. Scenario Analysis

#### 3.3.1. Changing Infection Rate

It is notable that before the implementation of the vaccination program in Hong Kong, the pandemic was brought well under control through strict intervention measures, and there was a low infection rate. The objectives of the vaccination program were to improve the immunity of the population to SARS-CoV-2, prepare for the gradual relaxation of social control measures, and drive economic recovery. As the cost-effectiveness result was sensitive to the infection rate variable, we set several scenarios with different levels of infection rate to simulate the difference in the cost-effectiveness ratio under different severities of the pandemic. Scenario A was the actual level of infection in Hong Kong before the vaccination program, while Scenarios B–K assumed higher infection rates of 0.25%, 0.5%, 1%, 1.2%, 1.5%, 2%, 5%, 10%, 20%, and 30%, respectively.

In the context of strict control measures and if the infection rate was maintained at a low level over a 1-year period, vaccination was not cost-effective. However, the cost-effectiveness of vaccines changed greatly when the infection rate changed. For Hong Kong, the vaccination program became cost-effective when the overall infection rate increased to 1%, and when it increased to 1.5%, the vaccination program became overwhelmingly worthwhile. Similarly, in the fifth wave, the vaccine program remained cost-effective despite the shift in treatment measures and became even more cost-effective as vaccine infection rates continued to rise (Table 3).

#### 3.3.2. Changing Protection Rate

As we have mentioned, after vaccination, the rate of protection decreases over time. In the general modeling process, we set the effectiveness of both vaccines to remain stable up to 6 months (91.3%) and then decrease by half (45.7%) in the following 6 months. Furthermore, as the virus mutates, the protection rate of existing vaccines will also change. In the model under the Omicron wave, we set the protection rate against mild infection adjusted to 16.5%. The range of vaccine protection rates against mild infection has exceeded the 25% of its expected value range in the sensitivity analysis. To ascertain the difference of cost-effectiveness of vaccination program under the change of vaccine protection rate in patients with mild disease, we conducted an additional scenario analysis on the change of protection rate.

Under the Omicron wave, based on the protection rate against mild infection dropping to 16.5%, the model estimated that the ICER of the vaccination program was HKD 310,094. If the vaccine’s protection against mild cases was assumed to remain unchanged at the original 91.3%, the estimated ICER decreased to HKD 96,877 (Table 4). As we see, the protection rate of the vaccine against mild disease is lower, the ICER value of the vaccine program is higher. At the willingness-to-pay threshold set for Hong Kong, even though the protection rate against mild disease is reduced to 16.5%, the vaccination program was still cost-effective.

#### 3.3.3. Changing Vaccination Rate

To ascertain the difference of cost-effectiveness of vaccination program under the change of vaccination rate, we conducted a scenario analysis on the change of vaccination rate. Scenario I was the actual level of vaccination rate in Hong Kong, while Scenarios P and Q assumed higher vaccination rate of 80% and 90%, respectively. Under the Omicron wave, the model estimated that the ICER of the vaccination program with vaccination rate of 72% was HKD 310,094. If the overall vaccination rate increase to 80%, the estimated ICER decreased to HKD 288,384. If the overall vaccination rate increased to 90%, the estimated ICER decreased to HKD 279,290 (Table 5). As we see, as the vaccination rate is higher, the ICER value of the vaccine program is lower, and the Vaccination Program is more cost effective.

#### 3.3.4. Summary of Scenario Analysis

**Figure 4:**
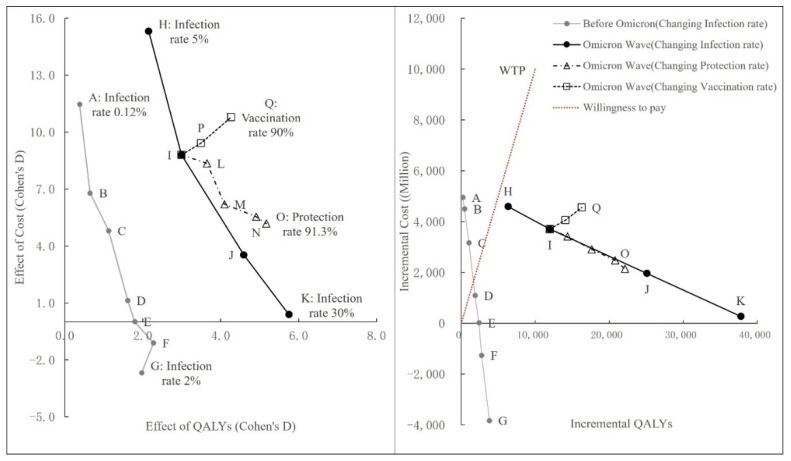
C–E plane of vaccination program of Hong Kong with scenarios in different infection rates, protection rates, and vaccination rates. (Point A–G show scenarios in different levels of the infection rate before Omicron wave. Point H–K show scenarios in different levels of the infection rate under Omicron wave. Point I, L–O show scenarios in different protection rate. Point I, P–Q shows scenarios in different vaccination rate.)

We have analyzed scenarios with different infection rates, protection rates, and vaccination rates. Figure 4 shows the cost-effective plane of the vaccination program of Hong Kong. The figure on the left shows effect size of vaccine program in scenarios, and the figure on the right shows the change of ICER value in scenarios. In general, it reveals that, as the epidemic rate increases, the vaccine program is always more cost-effective, both during the previous phase of tight containment (Scenario A–G) and during the Omicron phase of changing treatment and containment pattern (Scenario H–K). The effect of increased protection rate on the cost-effectiveness of the vaccine program is positive. As the protection rate of the vaccine against mild disease is higher, the vaccine program is more cost-effective (Scenario I, L–O). In addition, higher vaccination rates also have a positive effect on vaccination program. The vaccine program is consistently cost-effective, as increased vaccination rates lead to more effects, even if there is a cost involved (Scenario I, P–Q).

## 4. Discussion

### 4.1. Main Findings

We examined the cost-effectiveness of the vaccination program in Hong Kong. Under the strategy of strict control measures against the spread of COVID-19, the promotion of the vaccination program was not cost-effective before the Omicron wave. However, with the fifth wave of the Omicron variant, infection rates in Hong Kong have skyrocketed. In this case, the vaccine program became cost-effective. This conclusion is in keeping with other research on the topic. Research in the United States has shown that the COVID-19 vaccination program is predicted to provide good value for money, as vaccination is far superior to doing nothing, as it provides a greater number of QALYs at a lower cost [54]. Compared with no vaccination, the incremental cost per QALY gained by the adult population of the United States was USD 8200 [50]. Related studies in other regions have also arrived at a similar conclusion [55,56]. The main reason for the difference in the cost-effectiveness of vaccination programs between Hong Kong and other regions is that the pandemic background varies widely among regions. The United States and other Western countries have mostly taken less-stringent control measures, and the spread of the disease has been greater. Hong Kong kept the number of cases at a low level for a long period before the vaccination program began, but is also suffering a lot due to the Omicron variant. As the scenario analysis showed, if the pandemic becomes more severe (infection rates increase), there will be a greater opportunity for the vaccine to work, resulting in fewer cases and deaths.

Sensitivity analysis indicated that the BioNTech vaccine is more cost-effective than the Sinovac vaccine in Hong Kong, due to a higher vaccination rate and greater vaccine efficacy. For greater cost-effectiveness, future vaccination programs in Hong Kong could focus on the BioNTech vaccine. Vaccination rates among elderly individuals have the greatest effect on the cost-effectiveness of vaccination programs; thus, we propose that vaccination rates should be increased, especially among elderly individuals, to prepare for future outbreaks.

### 4.2. Limitations

This study represents an initial attempt to understand the burden of vaccination program on the Hong Kong economy. Our study, however, has some limitations. First, we did not include productivity losses for close contacts of infected individuals. This would understate the value of the vaccine, as productivity losses may be higher in the scenarios without the vaccine. Second, we did not include medicines in the model. Optimal supportive care includes oxygen for severely ill patients and those who are at risk of severe disease and more advanced respiratory support such as ventilation for patients who are critically ill. Additionally, WHO does not recommend self-medication with any medicines, including antibiotics, as a prevention or cure for COVID-19. Scientists around the world are working to find and develop treatments for COVID-19. It would be interesting to conduct studies on the cost-effectiveness of medicine treatments for COVID-19, if such medicines do become available. Third, we have been emphasizing that vaccine programs are cost-effective to contain the epidemic. However, this paper neglects to discuss issues about vaccination acceptance and vaccination hesitancy. In Hong Kong, the “Vaccination Pass” requires those entering or staying at designated premises to have been vaccinated, which encouraged citizens to get vaccinated. It is worth investigating whether there are other ways to address the issue of vaccination acceptance and hesitancy. This paper focused on the cost-effectiveness of the vaccine program, so there is no in-depth analysis of the vaccine hesitancy. The topic about vaccine acceptance and vaccine hesitancy has been analyzed by relevant studies [57,58,59], which can be referred to. Forth, our model mainly focused on cost-effectiveness from the perspective of the health sector. However, the costs and benefits associated with pandemic control measures involve all aspects of society, while our study was limited to evaluating the cost-effectiveness of the vaccination program only from the perspective of the health sector. In future studies, with greater knowledge of the long-term protection provided by vaccines, the cost-effectiveness of the COVID-19 vaccines with longer time horizons should be investigated to understand the longevity of the immune response. A cost-effectiveness analysis of non-vaccine interventions to estimate the cost burden of non-pharmacological intervention policies is also necessary. Moreover, the effectiveness of a mix of vaccine and non-vaccine interventions should be further investigated.

### 4.3. Implications

In the year prior to the implementation of the vaccination program in Hong Kong (February 2020–February 2021), the transmission rate was approximately 0.12%. Considering the Omicron wave in Hong Kong (December 2021–February 2022), the transmission rate was approximately 10% until now. United States has a population of 330 million [60]. There have been 78.4 million cases [1], thus the average annual infection rate is approximately 23.8%. The two-dose vaccination rate in the United States has reached 64.9% [61]. Taiwan, an area with relatively good control of the pandemic, has an average annual infection rate of approximately 0.09%, with 20,999 cases [62] from a population of 23.4 million [63]. The two-dose vaccination rate in Taiwan has currently reached 75.6% [64].

As our results revealed, the cost-effectiveness of a COVID-19 vaccine is sensitive to the infection rate. For regions with low prevalence of COVID-19, such as Hong Kong (before the Omicron wave) and Taiwan, which have managed to contain the disease well before the vaccination program was rolled out, non-pharmacological interventions have made a significant contribution to local epidemic control. If the government does not adjust the strict epidemic prevention and control measures, then the vaccination program will not be cost-effective in the short term. For regions with a high infection rate, faster and more widespread vaccination is the sensible option.

Hong Kong is transitioning from a low-infection area to a high-infection area. It is recognized that, even though the vaccine provides insufficient protection against mild cases, the vaccination program is still cost-effective. What is the point of a vaccination program in Hong Kong if it is not cost-effective in in the context of the low infection rate? First, the Hong Kong vaccination program is designed to deal with the potential risk of a resurgence of COVID-19 in the future, not to control the current outbreak. Second, vaccination is intended to boost the immunity of the local population in preparation for subsequent deregulation and future economic recovery. Therefore, we argue that there are limitations in discussing the cost-effectiveness of vaccines in the short term for Hong Kong. A longer-term strategic discussion about the significance of vaccination is much more consistent with the objectives of the vaccination program in Hong Kong. Furthermore, there are many uncertainties about how the pandemic will develop in the future, as changes in policy and individual behavior may affect the course of the disease, such that longer-term estimates are highly uncertain [65,66]. The ongoing outbreak of the Omicron virus in Hong Kong helps support this.

## 5. Conclusions

Understanding the cost-effectiveness of vaccines is imperative to better comprehend the value-for-money provided by vaccines. The promotion and implementation of the Hong Kong government’s vaccination program was estimated to decrease the number of infections and deaths. Thus, the vaccination program was effective at helping to contain the outbreak. However, the cost of the vaccination program is high. Based on the pandemic situation before the Omicron wave, it is estimated that the ICER of the vaccination program for the 1-year period from February 2021 to February 2022 was HKD 22,339,700. Based on the willingness-to-pay threshold for Hong Kong, the vaccination program was not cost-effective before the outbreak of Omicron.

The cost-effectiveness of a COVID-19 vaccine, however, is sensitive to the infection rate. New variants of the virus, reduced vaccine effectiveness, or the gradual easing of containment measures to revive the economy will bring uncertainty about the future spread of the pandemic. Hong Kong is now experiencing the fifth wave of the Omicron epidemic. The current annual infection rate in Hong Kong is at approximately 10%, and the number continues to rise. Omicron has vastly increased the number of patients, and Hong Kong’s medical system is failing to accommodate such a large number of patients in such a short time. Thus, many patients with mild symptoms have been isolated at home. It is estimated that the ICER of the vaccination program for the 1-year period from February 2022 to February 2023 was HKD 310,094. At the willingness-to-pay threshold set for Hong Kong, the vaccination program in Hong Kong has thus been cost-effective in the context of Omicron.

In general, the vaccination has played an effective role in reducing the infection and mortality rates, while also bringing a large economic burden; therefore, we must acknowledge the limitations of the vaccination program. This, in turn, will help relevant departments implement infection control measures, including vaccination programs and other measures. From a longer-term perspective, the vaccination program will demonstrate great cost-effectiveness once infection rates are high in a regional outbreak. For Hong Kong residents, vaccines and health care provided through public hospitals is free, with all expenses borne by the government through public taxation. As a result, residents’ perceptions of the cost-effectiveness of the vaccines are not sensitive, which has been helpful for the implementation of the vaccination program in Hong Kong [67]. Compared with other age groups, the elderly population is more sensitive to the cost-effectiveness of the vaccination program; thus, we recommend that the elderly population be prioritized to improve vaccine coverage rate.

## Figures and Tables

**Table 2:** Incremental cost-effectiveness ratio of the COVID-19 vaccination program in Hong Kong.

Scenario			Cost (HKD Billion)	Outcome			ICER		
Infection Rate	Comparators			Cases	Death	QALYs	HKD/Case	HKD/Death	HKD/QALYs
0.12% (Before Omicron)		No Vaccine	0.84	9630	269	7,393,955			
		Vaccination Program	5.80	3704	67	7,394,177	836,364	24,533,333	22,339,700
10.0% (Omicron Wave)	Lower Protect	No Vaccine	85.1	953,215	25,053	7,358,898			
		Vaccination Program	73.8	831,656	8620	7,372,388	−31,468	−687,705	−837,699
	Home quarantine	No Vaccine	5.1	954,158	23,975	7,359,845			
		Vaccination Program	7.4	355,385	8688	7,381,766	−6388	146,696	102,303
	Home quarantine and Lower Protect	No Vaccine	5.1	948,703	24,043	7,360,067			
		Vaccination Program	8.8	827,682	9092	7,372,033	−31,608	248,198	310,094

**Table 3:** Incremental cost-effectiveness ratio of the COVID-19 vaccination program adjusted for different levels of the basic infection rate in Hong Kong.

Scenario			Cost (HKD Billion)	Outcome			ICER			Cohen’s D	
Basic Infection Rate		Comparators		Cases	Death	QALYs	HKD/Case	HKD/Death	HKD/QALYs	Cost	QALYs
A	0.12%	No Vaccine	0.8	9630	269	7,393,955					
		Vaccination Program	5.8	3704	67	7,394,177	836,364	24,533,333	22,339,700	11.5	0.4
B	025%	No Vaccine	1.6	15,422	539	7,393,519					
		Vaccination Program	6.2	5657	202	7,393,962	461,379	13,380,000	10,153,029	6.8	0.7
C	0.5%	No Vaccine	3.6	40,273	1145	7,392,690					
		Vaccination Program	6.8	14,681	404	7,393,740	123,684	4,272,727	3,013,910	4.8	1.1
D	1%	No Vaccine	6.9	77,717	2424	7,391,470					
		Vaccination Program	8.0	29,093	875	7,393,334	22,576	708,696	588,990	1.1	1.6
E	1.2%	No Vaccine	8.6	97,315	2559	7,390,538					
		Vaccination Program	8.7	35,626	943	7,392,934	17,795	12,500	8431	0.0	1.8
F	1.5%	No Vaccine	10.6	118,529	2829	7,390,072					
		Vaccination Program	9.3	43,910	808	7,392,831	−16,877	−623,333	−456,513	−1.1	2.3
G	2%	No Vaccine	14.5	160,216	4175	7,388,097					
		Vaccination Program	10.6	59,669	1549	7,391,899	−38,044	−1,456,410	−1,006,250	−2.7	2.0
H*	5%	No Vaccine	2.6	475,867	12,392	7,376,796					
		Vaccination Program	7.2	415,323	4512	7,383,127	−63,181	582,906	725,490	15.3	2.2
I*	10%	No Vaccine	5.1	948,703	24,043	7,360,067					
		Vaccination Program	8.8	827,682	9092	7,372,033	−31,608	248,198	310,094	8.8	3.0
J*	20%	No Vaccine	10.2	1,885,755	49,499	7,325,343					
		Vaccination Program	12.2	1,646,812	17,443	7,350,423	−16,009	61,345	78,411	3.5	4.6
K*	30%	No Vaccine	15.1	2,795,734	73,138	7,291,951					
		Vaccination Program	15.3	2,448,094	24,985	7,329,714	−11,003	5734	7312	0.4	5.8

Note: Scenario H*, I*, J* and K* were simulated under the Omicron wave with lower protection rate against mild disease and home quarantine.

**Table 4:** Incremental cost-effectiveness ratio of the COVID-19 vaccination program adjusted for different levels of the protection rate under Omicron wave in Hong Kong.

Scenario			Cost (HKD Billion)	Outcome			ICER			Cohen’s D	
Efficacy for Mild		Comparators		Cases	Death	QALYs	HKD/Case	HKD/Death	HKD/QALYs	Cost	QALYs
I	16.5%	No Vaccine	5.1	948,703	24,043	7,360,067					
		Vaccination Program	8.8	827,682	9092	7,372,033	−31,608	248,198	310,094	8.8	3.0
L	30.0%	No Vaccine	5.2	941,834	23,504	7,360,363					
		Vaccination Program	8.5	723,229	8553	7,374,733	−17,498	228,378	237,605	8.4	3.7
M	45.6%	No Vaccine	5.2	953,821	24,716	7,359,623					
		Vaccination Program	8.1	601,871	8082	7,377,233	−10,869	174,899	165,213	6.2	4.1
N	70.0%	No Vaccine	5.2	947,558	25,996	7,358,802					
		Vaccination Program	7.7	462,600	9092	7,379,585	−7888	146,614	119,251	5.6	4.9
O	91.3%	No Vaccine	5.1	951,060	24,851	7,359,934					
		Vaccination Program	7.3	33,4036	9024	7,382,040	−6200	135,319	96,877	5.2	5.2

**Table 5:** Incremental cost-effectiveness ratio of the COVID-19 vaccination program adjusted for different vaccination rate under Omicron wave in Hong Kong.

Scenario			Cost (HKD Billion)	Outcome			ICER			Cohen’s D	
Vaccination Rate		Comparators		Cases	Death	QALYs	HKD/Case	HKD/Death	HKD/QALYs	Cost	QALYs
I	72%	No Vaccine	5.1	948,703	24,043	7,360,067					
		Vaccination Program	8.8	827,682	9092	7,372,033	−31,608	248,198	310,094	8.8	3.0
P	80%	No Vaccine	5.2	950,791	25,053	7,359,512					
		Vaccination Program	9.5	815,223	7004	7,373,594	−28,217	225,000	288,384	9.4	3.5
Q	90%	No Vaccine	5.2	947,356	25,120	7,359,453					
		Vaccination Program	9.7	798,589	4512	7,375,754	−25,713	220,915	279,290	10.8	4.3

## Data Availability

Not applicable.
